# Diagnostic use of fluorescence *in situ* hybridization in expert review in a phase 2 study of trabectedin monotherapy in patients with advanced, translocation-related sarcoma

**DOI:** 10.1186/s13000-016-0486-2

**Published:** 2016-04-12

**Authors:** Shintaro Sugita, Hiroko Asanuma, Tadashi Hasegawa

**Affiliations:** Department of Surgical Pathology, Sapporo Medical University, School of Medicine, South 1, West 16, Chuo-ku, Sapporo, Hokkaido 060-8543 Japan

**Keywords:** Fluorescence *in situ* hybridization, Translocation-related sarcoma, Immunohistochemistry, Synovial sarcoma, Malignant peripheral nerve sheath tumor, Sarcomatoid carcinoma

## Abstract

**Background:**

Fluorescence *in situ* hybridization (FISH) is one of the most powerful genetic analysis tools for pathological diagnoses. FISH can detect various genetic abnormalities including gene translocation that was specifically found in translocation-related sarcomas (TRSs). Here, we report the use of FISH in expert review in a phase 2 study of trabectedin monotherapy for patients with advanced TRS.

**Methods:**

TRS patients (*n* = 76) were enrolled in the trial at 12 study sites after pathological diagnoses were made, including morphological examination with or without evidence of translocation by genetic testing. Following histological reviews of the representative specimens at the study sites, we performed immunohistochemistry using the appropriate antibodies and FISH for genetic confirmation of the tumor types in the expert review.

**Results:**

Among the 76 TRS cases, no split signal for SS18 probe was detected by FISH in three synovial sarcoma cases that were diagnosed at the study sites. Malignant peripheral nerve sheath tumor (MPNST) was diagnosed in two cases and sarcomatoid carcinoma in one. One of the cases was a small round cell variant of MPNST. After excluding these three cases, we assessed the other 73. There were no split signals detected in 7 of the 73 cases by FISH analysis, due to decalcification and hyperfixation procedures. Excluding these seven cases, FISH detected translocations in 95 % (63/66) of the study cases with a high sensitivity.

**Conclusions:**

The diagnosis of TRS by FISH was highly sensitive and enabled genetic confirmation of the pathological diagnoses. We strongly recommend FISH as a confirmatory diagnostic test for TRS, which would enable the selection of patients with TRS in whom trabectedin is expected to be effective.

This study was done in part that registered with Japan Pharmaceutical Information Center, number JapicCTI-121850.

## Background

In general, sarcomas can be divided into two groups that comprise translocation-related sarcomas (TRSs) and non-TRSs [1]. The TRS group has a specific chimeric gene derived from a chromosome translocation inherent in specific tumor types. Fluorescence *in situ* hybridization (FISH) is one of the most powerful genetic research tools for making a pathological diagnosis [2]. FISH using routine slide glass specimens can detect various genetic abnormalities including gene translocation that gives rise to some chimeric genes, so it is suitable for genetic confirmation of TRSs.

Recently, Kawai et al reported the efficacy of trabectedin monotherapy after chemotherapy versus best supportive care in patients with advanced TRS [3]. Trabectedin is a tetrahydroisoquinoline alkaloid that has anti-tumor activities in soft tissue sarcomas and binds to the minor groove of DNA and blocks DNA repair machinery [4]. Preclinical data have shown that trabectedin also modulates the transcription of the oncogenic fusion proteins of TRS and some retrospective clinical studies have revealed that it is effective in TRS patients [5–7]. The trial was performed as a randomized, open-labeled, phase 2 study and demonstrated that trabectedin significantly reduced the risk of disease progression and death in patients with advanced TRS after standard chemotherapy. Trabectedin was recently approved in Japan after taking these results into consideration. We took charge of the expert review of all cases to confirm the primary pathological diagnosis obtained from each study site. In addition to morphological examinations and immunohistochemistry (IHC), we performed FISH, a practical and effective method, to obtain genetic confirmation of TRS diagnosis. To date, there are no detailed reports on the use of FISH for the genetic analysis of TRS cases in clinical trials. Here, we report the use of this method for genetic investigations in a clinical trial.

## Methods

### Case selection and relevant immunohistochemistry in expert review

TRS patients (*n* = 76) were enrolled in a trial involving 12 study sites after pathological diagnoses had been made, which were based on morphological examinations with or without evidence of translocation on genetic investigation. The 76 TRS patient were diagnosed as follows: 24 myxoid liposarcomas (MLS), 21 synovial sarcomas (SS), 6 mesenchymal chondrosarcomas (MCS), 5 extraskeletal Ewing sarcomas (EES), 5 alveolar soft part sarcomas (ASPS), 5 alveolar rhabdomyosarcomas (ARMS), 5 clear cell sarcomas (CCS), 2 extraskeletal myxoid chondrosarcomas (EMC), 1 dermatofibrosarcoma protuberans (DFSP), 1 angiomatoid fibrous histiocytoma (AFH), and 1 desmoplastic small round cell tumor (DSRCT). IHC had already been conducted at each study site using appropriate antibodies for pathological diagnosis before the expert review commenced. We reviewed the histology of the representative specimens at the study sites and performed IHC with relevant antibodies in expert review to estimate the diagnostic consistency at the study sites before genetic confirmation of the tumor types by FISH. The following antibodies were used to confirm the tumor types in the expert review: vimentin (V9; Dako, Carpinteria, CA), S-100 (polyclonal; Dako), MDM2 (Ab-1; Calbiochem, Darmstadt, Germany), and CDK4 (DCS-31; Invitrogen, Carlsbad, CA) for MLS; vimentin, bcl-2 (Bcl2; Dako), cytokeratin (AE1/AE3; Dako), epithelial membrane antigen (EMA) (E29; Dako), and c-kit (c-kit; Dako) for SS; vimentin, MIC2 (CD99) (12E7; Dako), S-100 for MCS; vimentin, MIC2, synaptophysin (SY38; Dako), and neurofilament (2 F11; Dako) for EES; TFE3 (polyclonal; Santa Cruz Biotechnology, Santa Cruz, CA) and vimentin for ASPS; desmin (D33; Dako), muscle-specific actin (HHF35; Dako), myogenin (F50; Dako), and vimentin for ARMS; vimentin, S-100, melanosome (HMB-45; Dako), and SOX-10 (polyclonal; Santa Cruz Biotechnology) for CCS; vimentin, S-100, NSE (polyclonal; Dako), and synaptophysin for EMC; CD34 (QBEnd10; Dako), vimentin for DFSP; vimentin, desmin, CD68 (PG-M1; Nichirei, Tokyo, Japan), MIC2, and EMA for AFH; and cytokeratin, EMA, desmin, and synaptophysin for DSRCT. Both the study protocol and the informed consent form were approved by the institutional review board at each study site. All patients gave written informed consent for enrollment into the clinical trial and the expert review [3].

### Fluorescence *in situ* hybridization

FISH was performed at SRL Medisearch Inc. (Tokyo, Japan) according to our routine procedure, which uses various commercial and in-house probes, as previously described [2]. We used dual-colored, split-signal probe sets for MLS, SS, EES, ASPS, ARMS, CCS, EMC, DFSP, AFH, and DSRCT, and in-house dual-colored, fusion-signal probe sets for MCS cases (Table 1). To estimate the rate of split signals, “split” was defined when the distance between the orange and green signals was as least twice that of the estimated signal diameter. We counted 50 nuclei and considered each case to be either positive, negative, or indeterminate if the split signals were observed in more than 25 (50 %) tumor cells, less than 2 (4 %) tumor cells, or 2 to 24 (4 to 48 %) tumor cells, respectively. Second, 50 nuclei in indeterminate cases were counted by another observer and the case was considered positive if the split signals were found in more than 5 (10 %) tumor cells. The rate of fusion signals was defined when orange and green signals completely overlapped, producing a yellow signal. We first counted 100 nuclei and considered each case as positive, negative, or indeterminate if the fusion signals were observed in more than 50 tumor cells (50 %), less than 10 (10 %) cells, and 10 to 49 (10 to 49 %) cells, respectively. In indeterminate cases, 100 nuclei were counted by another observer and the cases were considered positive if fusion signals were found in more than 21 (21 %) tumor cells. FISH signals were estimated on a Nikon ECLIPSE E600 microscope (NIKON CORPORATION, Tokyo, Japan) at 100× magnification with oil immersion, using a DAPI/green/orange filter set. The results of FISH were confirmed by T.H.

**Table 1:** List of FISH probes used in the expert review

Histological type	Probe
Myxoid liposarcoma	DDIT3 Dual Color Break Apart Probe (Abbott Molecular Inc., IL)
Synovial sarcoma	SS18 Dual Color Break Apart Probe (Abbott Molecular Inc.)
Mesenchymal chondrosarcoma	HEY1-NCOA2 fusion probe (Chromosome Science Lab Inc., Sapporo, Japan)
Extraskeletal Ewing sarcoma	EWSR1 Dual Color Break Apart Probe (Abbott Molecular Inc.)
Clear cell sarcoma	
Angiomatoid fibrous histiocytoma	
Desmoplastic small round cell tumor	
Alveolar soft part sarcoma	TFE3 split probe (Chromosome Science Lab Inc.)
Alveolar rhabdomyosarcoma	FOXO1 Dual Color Break Apart Probe (Abbott Molecular Inc.)
Extraskeletal myxoid chondrosarcoma	NR4A3 split probe (Chromosome Science Lab Inc.)
Dermatofibrosarcoma protuberans	PDGFB split probe (Chromosome Science Lab Inc.)
Angiomatoid fibrous histiocytoma	FUS Dual Color Break Apart Probe (Abbott Molecular Inc.)

## Results

**Fig 1:**
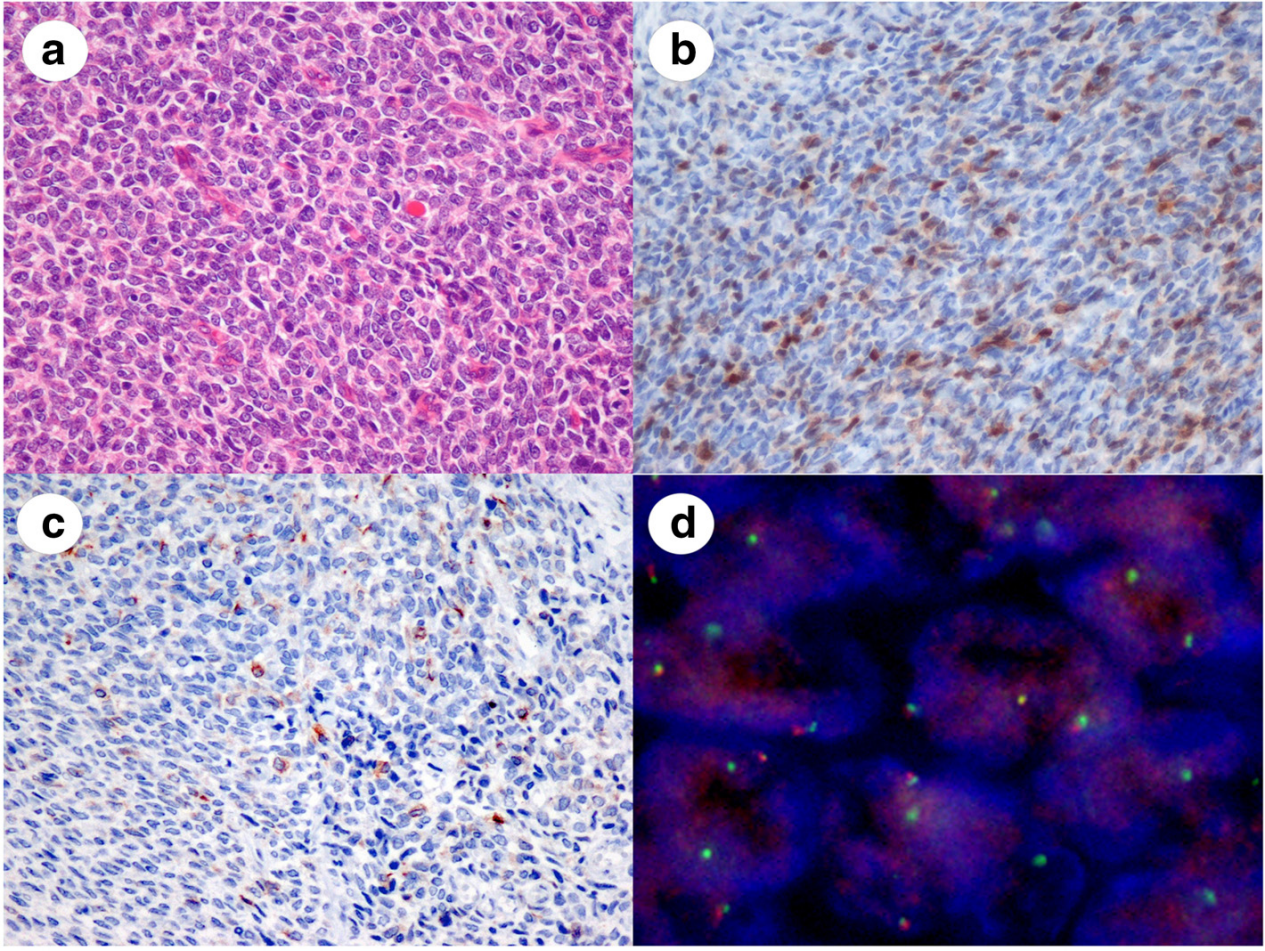
Representative images of a malignant peripheral nerve sheath tumor mimicking a poorly differentiated synovial sarcoma. **a**: The tumor consisted of solid proliferations of uniform, round tumor cells with round to oval nuclei. **b**: Tumor cells were focally positive for S-100 protein on IHC. **c**: Tumor cells were sparsely positive for cytokeratin AE1/AE3 on IHC, **d**: FISH revealed no SS18 split signals

The results of the confirmatory IHC tests in expert review largely supported the findings of the pathological diagnoses at the study sites, and there were no major discrepancies between morphological examinations and IHC results. We used FISH analysis to examine the 76 cases for genetic confirmation of pathological diagnoses (Table 2). Among these cases, SS18 split signals were not detected in three of the SS cases diagnosed at the study sites. MPNSTs were diagnosed in two cases and sarcomatoid carcinoma in one. The diagnosis of these three cases by morphology and IHC alone was challenging. One MPNST case showed solid proliferation of small round tumor cells but did not have the fascicular structure of spindle tumor cells, a typical MPNST feature (Fig. 1a). The tumor cells were diffusely positive for vimentin, focally positive for S-100 (Fig. 1b) and bcl-2, sparsely positive for cytokeratin (Fig. 1c), and negative for EMA and c-kit. Additional IHC revealed diffuse CD56 positivity. This case required differentiation from poorly differentiated SS, although SS18 split signals were not detected (Fig. 1d). The case was finally diagnosed as a round cell-type MPNST.

**Table 2:** Comparison of the pathological diagnosis of the study sites and expert review by FISH analysis

Histological type	Study site	Expert review	FISH		
			Positive	Negative	ND
Myxoid liposarcoma	24	24	22	0	2
Synovial sarcoma	21	18	16	4^a^	1^b^
Mesenchymal chondrosarcoma	6	6	3	0	3
Extraskeletal Ewing sarcoma	5	5	4	0	1
Alveolar soft part sarcoma	5	5	5	0	0
Alveolar rhabdomyosarcoma	5	5	4	1	0
Clear cell sarcoma	5	5	5	0	0
Extraskeletal myxoid chondrosarcoma	2	2	1	1	0
Dermatofibrosarcoma protuberans	1	1	1	0	0
Angiomatoid fibrous histiocytoma	1	1	1	0	0
Desmoplastic small round cell tumor	1	1	1	0	0
Total	76	73	63	6	7

^a^Four FISH-negative cases of synovial sarcoma included 2 MPNSTs, 1 sarcomatoid carcinoma, and 1 synovial sarcoma with *SS18-SSX* fusion detected by RT-PCR

^b^One FISH ND case with *SS18-SSX* fusion detected by RT-PCR

*ND*, not detected, *MPNST* malignant peripheral nerve sheath tumor

Another MPNST case consisted of fascicular proliferation of spindle tumor cells and showed typical spindle cell sarcoma features. IHC showed that these tumor cells were diffusely positive for vimentin, focally positive for cytokeratin and EMA, and negative for S-100, bcl-2, and c-kit. Additional IHC showed diffuse CD56 positivity. No SS18 split signals were observed and we finally diagnosed the case as ordinary MPNST.

**Fig 2:**
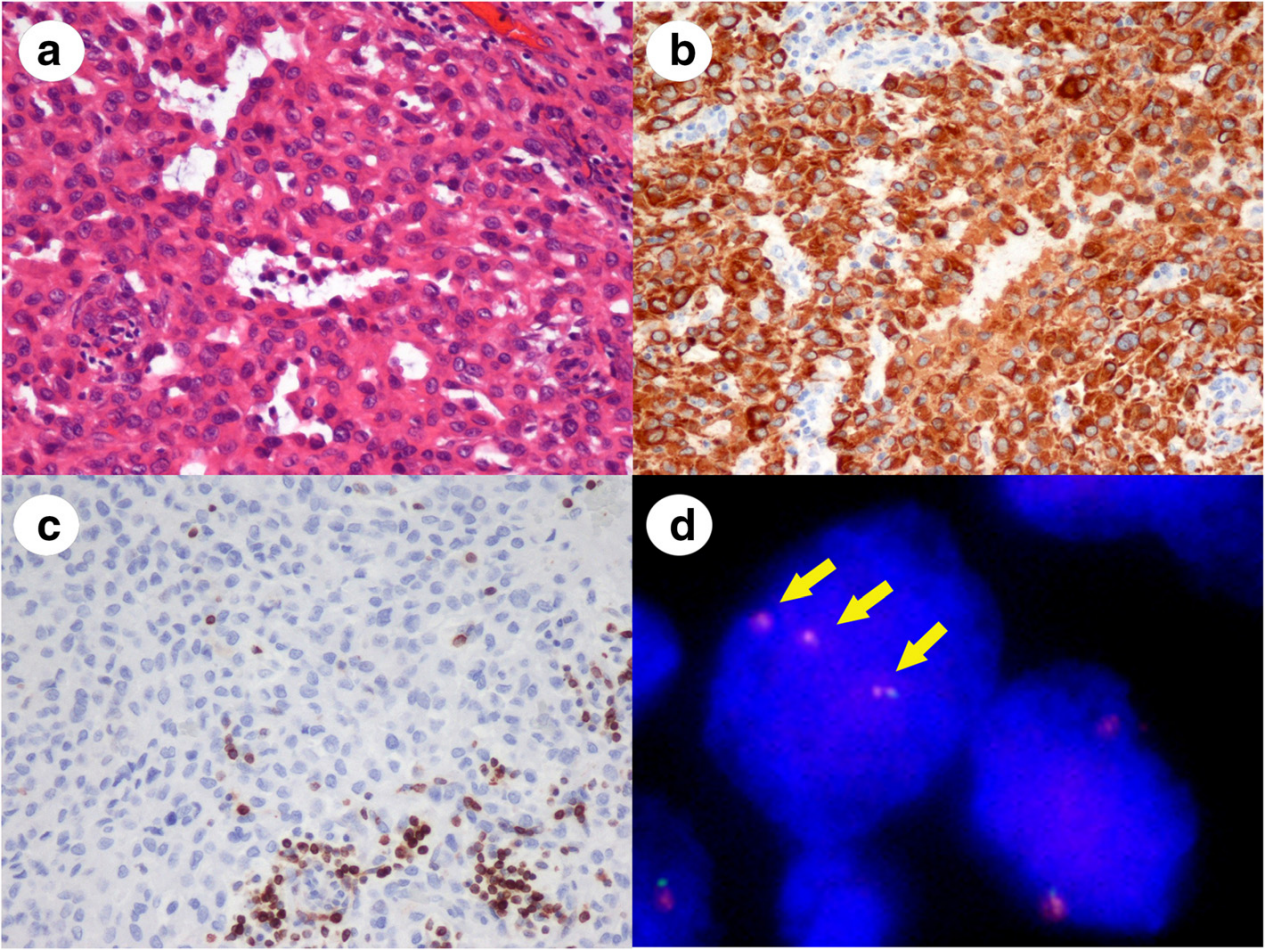
Representative images of sarcomatoid carcinoma after synovial sarcoma was excluded. **a**: The tumor had a pseudoglandular structure and a sheet-like proliferation of cuboidal tumor cells. **b**: Tumor cells were positive for cytokeratin AE1/AE3 on IHC. **c**: Tumor cells were negative for bcl-2 on IHC. Infiltrating lymphocytes alone were bcl-2 positive. **d**: FISH analysis revealed no SS18 split signals. Nuclei showed more than two pairs of orange and green signals with a polyploidy pattern (arrows)

In contrast, in the sarcomatoid carcinoma case, the tumor consisted of sheet-like and glandular proliferation of epithelioid tumor cells (Fig. 2a). The tumor cells were diffusely positive for cytokeratin (Fig. 2b), EMA, and vimentin, and negative for bcl-2 (Fig. 2c). The differential diagnosis in this case was epithelioid SS, but FISH detected a polyploidy pattern without SS18 split signals (Fig. 2d). We made a final diagnosis of sarcomatoid carcinoma.

**Fig 3:**
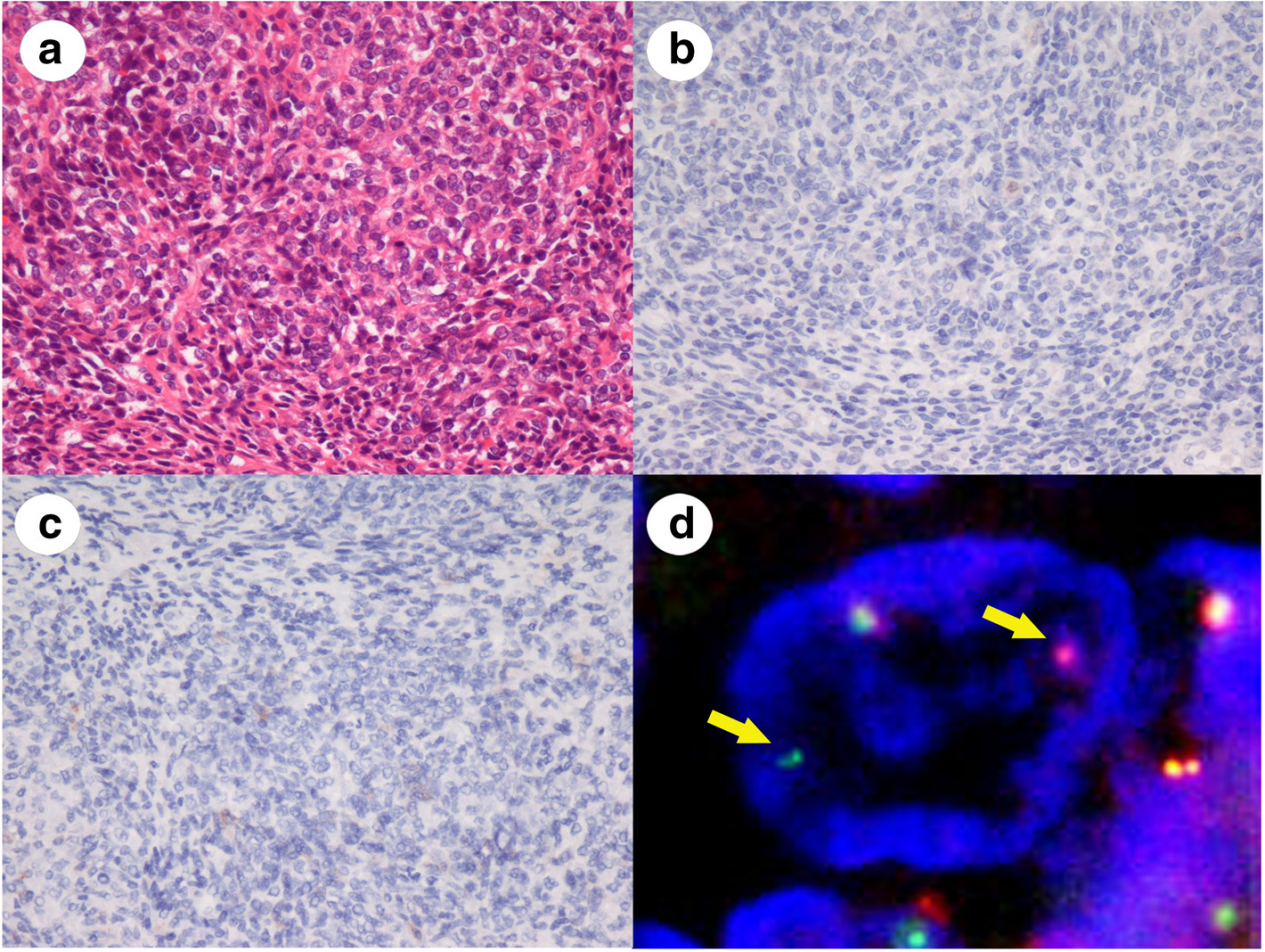
Representative images of poorly differentiated synovial sarcoma without cytokeratin expression. **a**: The tumor consisted of solid and fascicular proliferations of round to short-spindle tumor cells with round to oval nuclei. **b**: Tumor cells were negative for cytokeratin AE1/AE3 on IHC. **c**: Tumor cells were negative for EMA on IHC. **d**: FISH analysis revealed a pair of split and fused orange and green signals showing a SS18 split pattern (arrows)

The results of IHC corresponded to the histological diagnoses, although there was one SS case that demonstrated an unusual IHC staining pattern and was difficult to diagnose without FISH. The tumor consisted of solid proliferations of epithelioid and round tumor cells (Fig. 3a) and was similar to poorly differentiated SS, although no reactivity to cytokeratin and EMA was detected by IHC (Fig. 3b, c). FISH revealed SS18 split signals in the tumor cells and the case was diagnosed as SS (Fig. 3d). As mentioned above, the histological diagnoses of the 10 tumor types except for SS made at the study sites matched those of expert review.

We assessed the 73 TRS cases after excluding 3 cases. No signals were detected by FISH analysis in 7 of the 73 TRS cases. These cases included 2 MLS cases, 3 MCS cases, 1 SS case, and 1 EES case. One of the MLS cases had no nuclei due to karyorrhexis, and no green and orange signals were detected in 1 MLS case, 3 MCS cases, 1 SS case, and 1 EES case. These cases had undergone decalcification because they contained bony tissue. One EES was a minute biopsy specimen and showed formalin hyperfixation. Thus, the 7 cases with no FISH signals were inappropriate for FISH analysis. Excluding the 7 cases, FISH detected translocations in 95 % (63/66) of the 73 study cases with high sensitivity. In 1 SS case, SS18-SSX fusion was detected by RT-PCR, although no split signals were revealed by FISH. Moreover, one ARMS and one EMC case proved negative for FISH. Thus, it is thought that there were other chimeric fusions affecting these cases [2].

## Discussion

Morphological examination is undoubtedly the most fundamental and important procedure for pathological diagnosis. However, challenging cases require additional genetic investigations to confirm the diagnosis. In this study, we had some challenging cases in which the differential diagnosis needed to be made carefully. Two MPNST cases correctly diagnosed in expert review exhibited focal cytokeratin and EMA reactivity, confirming that distinguishing between SS and MPNST is essential. MPNST does not always express S-100 and occasionally has aberrant expression of epithelial markers, similar to the present cases [8, 9]. Moreover, MPNSTs express CD56; therefore, we suspected MPNST and confirmed the diagnosis by FISH. One of them was a small round cell variant MPNST that characteristically consists of small round tumor cells [10, 11]. This histological variant needs to be distinguished from poorly differentiated SS. Both tumors typically consist of solid proliferations of small round tumor cells and aberrant expression of epithelial markers, so care must be taken in reaching the differential diagnosis. SS sometimes shows no reactivity to epithelial markers, especially in monophasic and poorly differentiated cases [12, 13]. SS cases negative for epithelial markers must therefore be distinguished from various tumor types, given that confirmation of the diagnosis without detection of a specific rearrangement is challenging. We believe that the detection of a gene rearrangement by FISH could facilitate confirmation of the diagnosis in such situations.

FISH did not detect any signals in seven cases. These cases had undergone formalin hyperfixation and decalcification. Thus, these procedures might influence the condition of the specimens and prevent the detection of FISH signals. Specifically, the decalcification process markedly affects study results; our previous study demonstrated the negative effect of decalcification. We studied the use of FISH to detect HEY1-NCOA2 fusion in MCS cases; some cases of tumors originating in the bone showed no signals on FISH after the decalcification process [14]. In the present study, three MCS cases had no signals after decalcification, although a diagnosis of MCS was easily reached because of the characteristic histological findings.

## Conclusion

We performed FISH for genetic confirmation of pathological diagnoses in expert review during a clinical trial. FISH demonstrated a high sensitivity for TRS and its value as a genetic testing tool for clinical trials. We strongly recommend FISH as a confirmatory diagnostic test for TRS, which would enable the selection of patients with TRS who are expected to be effectively treated by trabectedin.

### Availability of supporting data

None.

## Abbreviations

IHC: immunohistochemistry
FISH: fluorescence *in situ* hybridization
TRS: translocation-related sarcoma
